# Antidiabetic Drugs in Alzheimer's Disease: Mechanisms of Action and Future Perspectives

**DOI:** 10.1155/2017/7420796

**Published:** 2017-06-01

**Authors:** Grazia Daniela Femminella, Leonardo Bencivenga, Laura Petraglia, Lucia Visaggi, Lucia Gioia, Fabrizio Vincenzo Grieco, Claudio de Lucia, Klara Komici, Graziamaria Corbi, Paul Edison, Giuseppe Rengo, Nicola Ferrara

**Affiliations:** ^1^Neurology Imaging Unit, Imperial College London, London, UK; ^2^Division of Geriatrics, Department of Translational Medical Sciences, Federico II University of Naples, Naples, Italy; ^3^Center for Translational Medicine and Department of Pharmacology, Lewis Katz School of Medicine, Temple University, Philadelphia, PA, USA; ^4^Department of Medicine and Health Sciences, University of Molise, Campobasso, Italy; ^5^Istituti Clinici Scientifici Maugeri SpA Società Benefit, Telese Terme Institute (BN), Italy

## Abstract

Diabetes mellitus (DM) and Alzheimer's disease (AD) are two highly prevalent conditions in the elderly population and major public health burden. In the past decades, a pathophysiological link between DM and AD has emerged and central nervous system insulin resistance might play a significant role as a common mechanism; however, other factors such as inflammation and oxidative stress seem to contribute to the shared pathophysiological link. Both preclinical and clinical studies have evaluated the possible neuroprotective mechanisms of different classes of antidiabetic medications in AD, with some promising results. Here, we review the evidence on the mechanisms of action of antidiabetic drugs and their potential use in AD.

## 1. Introduction

Diabetes mellitus (DM) represents a major public health burden and a growing prevalent chronic disease. It is known that more than 400 million have diabetes, and it is estimated that the number of diabetic patients is expected to rise to over 640 million by 2040 [1]. Alzheimer's disease (AD) is the main cause of dementia, affecting over 26 million people worldwide [2], and its prevalence continues to increase [3]. Both conditions are related to age, and in the last decades, an interesting link between the two diseases has emerged from various studies [4, 5]; thus, the term “type 3 diabetes” has been proposed to define insulin resistance-induced AD [6]. Many epidemiological evidence show an almost doubled risk for AD in diabetic patients, compared with nondiabetics [7]; the Rotterdam study showed a twofold increase of AD in DM and an even quadrupled risk associated with insulin therapy [8]. Although the pathophysiological connections are still not fully elucidated, two main key points have been identified to explain this association: insulin resistance and inflammatory signalling pathways [9].

## 2. DM and AD: Shared Pathophysiological Components

Hyperinsulinemia and insulin resistance, two of the hallmarks of type II DM (T2DM), have been shown to be important risk factors for elderly cognitive decline [10]. Indeed, while an acute administration of insulin may improve memory domains, dysfunctions in delayed memory process can result from chronic administration [9]. Insulin signalling induces brain to take up glucose and to produce insulin-degrading enzyme (IDE), in order to reduce its level. IDE is involved in both insulin and amyloid beta (A*β*) degradation; thus, hyperinsulinemia might determine a competitive inhibition for IDE-dependent A*β* degradation, leading to A*β* accumulation [11]. Moreover, in diabetes, alteration of insulin signalling determines less IDE production, resulting in reduction of A*β* degradation; the process definitely leads to abnormal A*β* accumulation in the brain. Therefore, increasing insulin signalling in the brain might reduce A*β* accumulation. Insulin has also been reported to enhance A*β* clearance from the brain [12]. Furthermore, soluble A*β* oligomers, known as amyloid beta-derived diffusible ligands (ADDLs), contribute to insulin resistance in AD by modifying synapse conformation. This altered shape conformation is responsible for reduced affinity of synaptic insulin receptor for its ligand [9].

Moreover, abnormal protein processing characterizes many neurodegenerative disorders. In particular, deposition of extracellular A*β* plaques appears to be exacerbated by impaired insulin signalling function in AD [13]; abnormal A*β* induces hyperphosphorylation of the tau protein, the major component of intracellular neurofibrillary tangles (NFT) [14]. These altered pathways involve glycogen synthase kinase-3 (GSK-3), the enzyme that phosphorylates tau to create AD neurofibrillary tangles, which has been shown to be downregulated in response to insulin [15]. Neuropathology of AD is characterized by loss of synapses, while insulin receptor signalling increases synaptic density. Interestingly, impairment of insulin signalling seems to precede A*β* accumulation in a transgenic mouse model of AD [16]. Furthermore, tau gets phosphorylated by c-Jun NH_2_-terminal kinase (JNK), which is activated by chronic hyperglycaemia and regulated by JNK-interacting protein 1, also known as “islet brain 1 protein” for its brain and pancreatic islet expression [17].

Both T2DM and AD are largely related to inflammatory processes. Insulin resistance is associated with elevated levels of proinflammatory cytokines such as C-reactive protein, tumor necrosis factor- (TNF-) *α*, interleukin- (IL-) 1, and IL-6 [18]. All these cytokines are considered an indirect sign of the immunological dysfunction that leads to insulin resistance [19]. Likewise, IL-6 and C-reactive protein are connected to A*β* plaque deposition and progression, and on the other side, a reduced AD incidence has been reported in patients under chronic nonsteroidal anti-inflammatory therapy [4]. Another relevant aspect is represented by the proinflammatory role of astrocytes and microglia surrounding A*β* plaques, that are responsible of neuronal irreversible damage as a consequence of complement cascade activation [20]. Interestingly, insulin seems to have anti-inflammatory effects directly suppressing proinflammatory cytokines and inducing anti-inflammatory mediators, as demonstrated in both preclinical and clinical studies [21].

Central obesity, defined as both high body mass index and mean waist circumference, represents a well-known risk factor for the development of insulin resistance, through an increased inflammatory response that alters insulin receptor signalling pathway. This may result in metabolic syndrome, a disorder also characterized by dyslipidaemia and hypertension, frequently precursor of T2DM. The role of obesity in promoting AD has been explored in many studies [22], and although the underlying mechanisms of this interaction is not yet known, AD risk is correlated with insulin resistance, oxidative stress, advanced glycation end products (AGEs), and hyperglycaemia. Furthermore, some evidence suggest that leptin could be used as a biomarker, in order to improve the understanding of AD risk and progression [23]. Epidemiological data suggest that insulin resistance is associated with increased risk of cognitive impairment [24], and PET studies have demonstrated that greater insulin resistance is associated with an AD-like pattern of reduced cerebral glucose metabolic rate in frontal, parietotemporal, and cingulate regions in adults with T2DM [25]. Thus, it is not surprising that insulin could be an effective treatment for AD by increasing neuronal glucose uptake and cellular ATP levels [26].

Dyslipidaemia and hypercholesterolemia contribute to the development of both DM and AD. Apolipoprotein E4 (APOE4), predominantly expressed in the liver and the brain, is the major genetic risk factor for sporadic AD [27]. In particular, APOE4 is associated with an increase in A*β* plaque deposition and susceptibility to oxidative stress [28], compared to the other APOE isoforms. Furthermore, higher rate of tau phosphorylation is induced by the overexpression of APOE4 in transgenic mice [29], while hippocampal expression of IDE is negatively correlated with APOE isoform 4 [11].

AGEs are heterogeneous compounds deriving from sugar irreversible nonenzymatic reactions with the protein amino groups, nucleotides, and lipids. This process that normally occurs during aging is accelerated in diabetic patients, due to the increased formation of reactive oxygen species. At this regard, Sasaki and collaborators have described an enhanced AGE immunoreactivity also in AD patients, particularly in A*β* plaques and NFT of hippocampal neurons [30].

The abovementioned overproduction of reactive oxygen species and the general increase in oxidative stress are characteristics of DM. Oxidized protein accumulation has also been demonstrated in the hippocampus, frontal and temporal lobes of mild cognitive impairment patients, suggesting an early impact of oxidative damage in AD development [14]. Mitochondria seem to play a pivotal role in this process as suggested by Moreira and colleagues, who have identified an involvement of mitochondrial dysfunction (oxidative phosphorylation uncoupling and respiratory chain alteration) in the development of neural degeneration and uncontrolled metabolism in rat model of T2DM [31].

## 3. Classes of Antidiabetic Drugs as Potential Treatments for Alzheimer's Disease

Given the multiple links between DM and AD, it is not surprising that several drugs currently approved for DM could also have a role in treating AD [32]. Different studies have explored the possible neuroprotective mechanisms of antidiabetic medications, and some of them have also been tested in clinical trials in AD and mild cognitive impairment (MCI) subjects. Here, we summarize some of the evidence from preclinical and clinical studies.

### 3.1. Insulin

Preclinical studies have shown that in the rat model of intracerebroventricular streptozotocin (STZ) injection-induced cognitive dysfunction, the intraventricular administration of detemir, a long-acting insulin analog, rescued STZ-induced cognitive decline, as evidenced by a significant elevation in learning ability. Moreover, detemir treatment resulted in changes in hippocampal levels of insulin receptor [33]. Early clinical studies have indicated that hyperinsulinemia without hyperglycaemia enhances memory in adults with AD, suggesting an important role of this hormone in memory facilitation [34]. However, the systemic administration of insulin is associated with increased risk of hypoglycemia and reduced penetration in the central nervous system (CNS). To overcome these issues, intranasal administration of insulin has been tested in several studies, proving to be effective in directly targeting the brain [35]. Preclinical evidence indicated that insulin administered intranasally bypasses the blood-brain barrier (BBB), and human studies have shown that following intranasal administration, a significant amount of insulin reaches the brain in a functionally active state [36].

In healthy volunteers, the intranasal administration of insulin (4 × 40 IU/d) for 8 weeks resulted in a significant improvement in delayed word recall tests and enhanced mood [37]. In a comparative study between regular human insulin and the rapid-acting insulin analog insulin aspart, it has been demonstrated that after 8 weeks of intranasal treatment, memory performance measured by word list recall was improved compared to placebo in both the aspart and the regular insulin groups. The aspart-treated subjects performed even better than those of the regular insulin-treated group [38]. Similar findings were observed in a group of obese subjects treated with intranasal insulin for 8 weeks: insulin treatment did not induce any significant reduction of body weight but declarative memory and mood were improved [39]. Insulin is also able to increase regional cerebral blood flow in the insular cortex and the putamen in healthy volunteers [40].

In patients with early AD and MCI, acute intranasal administration of 20 or 40 IU of insulin facilitated verbal memory recall especially in APOE4− subjects. Interestingly, memory-impaired APOE4+ subjects showed poorer recall following insulin administration, suggesting that APOE might have a role in mediating insulin effects in the CNS [41]. Further studies from the same group have also demonstrated that 21 days of intranasal insulin were able to improve attention, verbal memory, and functional status in AD and MCI subjects and to raise plasma concentrations of the short form of the beta-amyloid peptide, resulting in an increased A*β* 40/42 ratio [42]. The effects of insulin on cognition are dose-dependent, with maximal effect at 20 UI, and modulated by APOE genotype [43]. These data prompted the first pilot trial with intranasal insulin in 104 patients with AD and MCI. Treatment with 20 or 40 IU of insulin improved cognition and functional ability compared to placebo, as measured by ADAS-cog and ADCS-ADL, respectively. Moreover, cerebral 18F-fluorodeoxyglucose (18F-FDG) uptake significantly worsened at 4 months in placebo-treated group, while no changes in CSF biomarkers were observed [44]. Recently, the long-lasting insulin detemir has been tested for intranasal administration in AD and MCI, showing a treatment effect for the memory composite outcome compared with placebo. Similar to previous studies, this effect was moderated by the APOE status [45]. These preliminary data have prompted larger double-blinded placebo-controlled trials of insulin in AD. Two are ongoing in the US at the moment and are expected to complete recruitment in 2017 (NCT01767909; NCT02503501).

### 3.2. Metformin

Metformin is a biguanide that increases insulin sensitivity in peripheral tissues and suppresses hepatic gluconeogenesis. The evidence for its use in AD are controversial. Preclinical data suggest that orally administered metformin rapidly crosses the BBB and accumulates in CNS structures at different levels [46]. In vitro experiments on neuronal cell lines under prolonged hyperinsulinemic conditions have shown that treatment with metformin was able to resensitize insulin signalling and prevent the molecular and pathological changes observed in AD neurons [47]. Moreover, in murine primary neurons from wild type and tau transgenic mice, metformin treatment was able to reduce tau phosphorylation, one of the pathological hallmarks of AD [48]. In a murine model of leptin-resistant obese mice, systemic administration of metformin attenuated the increase of total tau and phospho-tau in the hippocampus but had no significant effects on tests of spatial learning and memory [49]. A population-based case-control study evaluating the incident risk of AD in diabetic patients on different antidiabetic medications suggested that overall the long-term use of sulfonylureas, thiazolidinediones, or insulin was not associated with an altered risk of developing AD. However, a slight increase of AD was reported in chronically metformin-treated patients [50]. Similarly, another study has reported that in patients with diabetes, use of metformin was associated with worse cognitive performance that could be attenuated by coadministration of vitamin B12 and calcium supplements [51]. Recently, the results of a 12-month pilot trial of metformin in 80 patients with MCI have shown that compared to placebo, significant changes were observed in verbal memory scores. Brain glucose uptake or plasma amyloid levels did not show significant differences in the two groups [52].

### 3.3. Thiazolidinediones

These drugs are agonists of the peroxisome proliferator-activated receptor-gamma (PPAR*γ*). In DM, they reduce serum glucose levels in response to insulin; moreover, they improve insulin resistance and ameliorate cholesterol homeostasis [53]. Currently, only pioglitazone is approved in DM therapy, while rosiglitazone has been withdrawn from the market due to high incidence of cardiovascular events. Both rosiglitazone and pioglitazone have been tested as potential treatment in AD, with inconclusive results. The potential for their use in AD derives from evidence showing an increased expression of PPAR*γ* in AD temporal cortex compared to controls [54]. Moreover, in preclinical studies, PPAR*γ* agonists have been shown to ameliorate AD-related pathology, probably reducing the expression of inflammatory genes and decreasing amyloid plaque burden [55]. Moreover, they seem to exhibit neuroprotective effects modulating calcium homeostasis in the hippocampus [56]. An early pilot study with rosiglitazone in AD and MCI patients demonstrated that 6 months of treatment were able to improve delayed recall and attention compared to placebo [57]. A larger study with three different doses of rosiglitazone (2, 4, or 8 mg for 6 months) showed that a significant improvement in the primary endpoint (ADAS-Cog change from baseline) was only observed in APOE4− patients on 8 mg [58]. However, larger phase III trials of rosiglitazone in AD as adjunctive therapy to acetylcholine esterase inhibitor (AChEI) failed to show any significant improvement in cognition or global function in patients stratified according to their APOE4 status [59].

Similarly, a pilot study with pioglitazone in AD patients with DM indicated that 15–30 mg of pioglitazone for 6 months improved cognition and cerebral blood flow in the parietal lobe compared to controls. Also, pioglitazone treatment resulted in a decrease in fasting plasma insulin levels, indicating enhanced insulin sensitivity [60]. Another pilot trial assessing pioglitazone safety in patients with AD without DM demonstrated that 18 months of treatment were well tolerated by patients but no significant data on efficacy were observed [61]. A recent metanalysis on PPAR*γ* agonists in AD suggests that only pioglitazone may offer an improvement in the early stages of AD and in mild-to-moderate AD [62]. Recently, it has been announced that the TOMORROW clinical trial, assessing the efficacy of 24 months of pioglitazone treatment in patients with MCI (NCT1931566), has completed its recruitment of 3500 subjects. The trial is also investigating a genetic-based biomarker risk assignment algorithm that comprises three components: APOE status, translocase of outer mitochondrial membrane (TOMM40) genotypes, and age. Its results will be available in 2019.

### 3.4. Glucagon-Like Peptide Receptor Agonists and Dipeptidyl Peptidase-4 Inhibitors

Glucagon-like peptide-1 (GLP-1) is a peptide hormone belonging to the incretin family, and it is secreted by the intestine in response to food intake. Its receptors (GLP-1Rs) can be found on pancreatic *β*-cells and, in response to high glucose levels, promote insulin release. Once secreted, native GLP-1 is degraded within minutes by the enzyme dipeptidyl peptidase-4 (DPP4); therefore, GLP-1 analogs that are resistant to DPP4 action have been developed for clinical use. These GLP1-R agonists (exendin-4, liraglutide, and lixisenatide) have been approved for treatment of DM [63]. However, GLP-1Rs are also present in the CNS, especially in the hypothalamus, hippocampus, cerebral cortex, and olfactory bulb [64]. GLP-1 seems to have several favorable effects within the CNS, where activation of GLP-1Rs protects against apoptosis, is neuroprotective against different stimuli, and induces neurite outgrowth, particularly in the hippocampus [65]. Several preclinical studies have tested the potential neuroprotective effects of GLP-1 analogs in AD, with promising results. Systemic administration of liraglutide for 8 weeks in AD transgenic mice prevented memory impairment, neuronal loss, and deterioration of synaptic plasticity in the hippocampus. Moreover, litaglutide was able to reduce amyloid plaque deposition by 40–50%, and reduce inflammatory response, as measured by activated microglial cells [66]. Similarly, in rats treated with intrahippocampal injection of A*β*, pretreatment with liraglutide significantly protected against the A*β*-induced impairment of spatial memory and long-term potentiation [67]. Further preclinical studies have demonstrated that liraglutide promotes neurogenesis, reduces tau hyperphosphorylation, and is also able to exert beneficial effects on cerebral and systemic microvasculature in AD transgenic mice [68–70]. Importantly, liraglutide does not only have preventive properties but can also reverse some of the key pathological hallmarks of late-stage AD in mice [71]. A pilot clinical trial testing liraglutide in AD patients has demonstrated that 6-month treatment with this GLP-1 analog was able to prevent decline of brain glucose metabolism, although no significant cognitive changes were observed compared to the placebo group [72]. A larger trial (ELAD) is currently ongoing at the Imperial College London, evaluating liraglutide in a large group of AD patients (NCT01843075). Among the other GLP-1 analogs, also, exenatide has shown promising results for its use in neurodegenerative diseases in preclinical studies [73], and a clinical trial on early-stage Alzheimer's disease or mild cognitive impairment is expected to publish their results soon (NCT01255163).

The inhibitors of DPP4, or gliptins, stabilize GLP-1 levels by inhibiting its degradation and lower fasting and postprandial glucose. Saxagliptin and vildagliptin have been approved in DM and have also been tested in preclinical studies of STZ-induced AD. Oral administration of both saxagliptin and vildagliptin resulted in attenuation of A*β* deposition, tau phosphorylation, and inflammatory markers and an improvement in hippocampal GLP-1 levels and memory retention [74, 75]. No clinical data are currently available for the potential effects of gliptins in AD patients.

### 3.5. Amylin Analog

Amylin is a small peptide hormone that is cosecreted with insulin from pancreatic *β*-cells in response to nutrient intake. It shares several features with A*β*, including similar *β*-sheet structure and being degraded by the insulin-degrading enzyme [76]. Amylin can cross the BBB and seems to have a role in the regulation of memory, mood, and anxiety [35]. However, because of its amyloidogenic potential, its analog pramlintide has been approved by the FDA, for use in DM type 1 and type 2. In AD patients, amylin plasma levels are significantly reduced and preclinical data in mouse models of AD suggest that pramlintide administration might improve memory, decrease oxidative stress, and reduce neuroinflammation [77]. Further studies are needed to assess the potential role of amylin and its analog in AD.

## 4. Conclusions

Of over 400 trials performed in AD from 2002 to 2012, only one has led to the approval of a medication (memantine in 2003) for its clinical use in AD, proving that drug development in AD is indeed very difficult [78]. Disappointing results have also been reported by the failure of antiamyloid strategies, suggesting that other pathways need to be explored in the search of disease-modifying agents in AD. The pathophysiological links between AD and DM and the availability of several antidiabetic drugs that have already shown in preclinical studies a potential beneficial effect on multiple aspects of neurodegeneration certainly suggest that these medications need further evaluation in AD. Encouraging results have been obtained by multiple clinical trials of intranasal insulin administration in AD, with no major side effects, making insulin a promising treatment in AD. Currently, several trials with antidiabetic drugs in AD and MCI are underway and their results are expected in the next few years. Besides, further studies exploring the molecular mechanisms underlying the favorable effects of antidiabetic drugs in the CNS could open new perspectives on future treatments.
